# Association of Happiness and Nursing Work Environments with Job Crafting among Hospital Nurses in South Korea

**DOI:** 10.3390/ijerph17114042

**Published:** 2020-06-05

**Authors:** Sujin Chang, Kihye Han, Yongae Cho

**Affiliations:** 1 Department of Nursing, Seoul National University Bundang Hospital, Gyeonggi-do 13620, Korea; zzanga0520@naver.com; 2 College of Nursing, Chung-Ang University, Seoul 06974, Korea; yacho2018@cau.ac.kr

**Keywords:** happiness, job crafting, nurses, work environment

## Abstract

Nurses are key professionals in healthcare sectors, whose job attitude is closely associated with patient health outcomes and safety. Job crafting describes how workers shape their tasks to find a sense of meaning and value in their work. This study aimed to examine the associations of happiness at the individual level and nursing work environments at the organizational level with job crafting among hospital nurses in Korea. This cross-sectional study analyzed survey data from 220 nurses working in four Korean hospitals. Multiple linear regression modeling was used to examine associations among the study variables. Nurses who were satisfied with their lives were significantly more likely to exhibit higher levels of job crafting (B = 0.07, *p* < 0.001). Nursing work environments had no significant association with nurses’ job crafting. In comparison with nurses working in general units, operating room nurses were significantly less likely to craft their job (B = −0.35, *p* = 0.001). Organizational support should be established to improve nurses’ happiness and job crafting. Hospitals should provide various opportunities for education and training to strengthen job crafting.

## 1. Introduction

Due to rapid technological changes and an explosive increase in information volume and flexibility in the workplace, job descriptions in the healthcare sector require revision and updating frequently [1]. Job-centered human resource management has expanded granular job units and the roles of individuals, leading to organizational changes. Currently, it is considered that change cannot occur without workers who voluntarily immerse themselves in these organizational changes [2]. Individual autonomy, creativity and immersion have come to the fore in terms of workforce management because these characteristics positively affect the organization and help achieve organizational goals. In these contexts, job crafting has attracted increased attention. Job crafting is defined as the process of modifying tasks to make the work more meaningful [3]. Job crafting is related to workers’ behaviors, i.e., their intention to find a sense of meaning and value in their work, and it also has a positive effect on both individuals and the organization. Previous research supports the positive results of job crafting, including job satisfaction and organizational commitment [4], psychological well-being [5] and productive job outcomes [6].

As nurses are the key professionals in healthcare sectors, their job attitudes are closely associated with patient health outcomes and safety [7]. In organizations where nurses deliver quality nursing care with positive attitudes toward their jobs, turnover and healthcare costs are reduced [8]. For these reasons, hospitals should understand factors associated with nurses’ job crafting and encourage nurses to develop positive motivation for their work. The key determinants of job crafting have been reported as individual characteristics such as a high sense of vocation [3], self-directedness [9], high levels of psychological capital [10], intrinsic motivation for job performance [11] and extroversion [12]. Workers can achieve job crafting through self-directed behaviors; thus, the effect of the personal characteristics of the job crafters themselves would be greater than that of organizational characteristics [6].

On a personal level, happiness is the ultimate goal in life for nearly everyone. As workers spend most of their daily lives in workplace settings, their happiness is closely related to their working life [13]. People who often experience happiness are more likely to actively set new goals at work and try to realize them. These factors, in turn, can result in better job outcomes [14]. Nurses who are happy can become immersed in their professional nursing practice, perform their work creatively and have a positive effect on organizational performance [15]. Happiness—a positive emotion—and internal satisfaction with life can act as intrinsic motivators for nurses to craft their jobs.

In terms of human resource management for nurses, organizations should foster good work environments in which job crafting can be emphasized and facilitated [3]. Nurses provide frontline care to patients and collaborate with various professionals in hospitals, and thus require frequent emotional exchanges with coworkers [16]. Due to high nurse turnover rates and low staffing levels, it may not be feasible for hospital nurses to undertake new tasks or additional work during their shifts [17]. It is important to create working environments where nurses can find the true meaning of nursing practice and support them with policies that engender positive motivation for their work.

Individual and organizational factors have rarely been collectively included as potential factors for job crafting in studies involving Korean nurses. Therefore, our study aimed to examine the effects of happiness at the individual level and nursing work environments at the organizational level on the job crafting of hospital nurses in Korea. Our research question was whether happiness and nursing work environments may be associated with the job crafting of hospital nurses in Korea.

## 2. Materials and Methods

### 2.1. Design

This study employed a cross-sectional survey design.

### 2.2. Participants and Data Collection

We selected four hospitals based on variations in hospital characteristics, which included teaching status (2 teaching and 2 non-teaching) and location (2 in Seoul and 2 outside metropolitan areas). All hospitals had more than 500 beds. After obtaining approval from the Institutional Review Board of the study hospital, data were collected in April 2019. Nurses with more than 6 months of nursing experience were invited to participate in the study, and approximately 2900 nurses in the four hospitals met the inclusion criteria. As the study units were conveniently selected, the number of available nurses was estimated at 450. A structured questionnaire along with the recruiting notice and informed consent form were enclosed in an envelope. We distributed 240 data collection packages. After completing the consent form and questionnaire, the participants were asked to enclose the materials in a sealed envelope and return them to designated locations for collection by the research staff. To maximize voluntary participation, anonymity and confidentiality, no hospital associates were involved in data collection procedures. Of the 240 questionnaires, 231 were returned (a return rate of 96%). After excluding 11 nurses with missing information for key variables, we analyzed the data for 220 nurses. According to Cohen’s (1988) formula for determining the appropriate sample size for multiple linear regression with an effect size of 0.15 (i.e., medium size), a power of 0.95 and an alpha of 0.05 with 12 explanatory variables, the study required a sample size of 184 [18]. Therefore, the sample size of our study was deemed sufficient.

### 2.3. Measures

Happiness was measured using the Korean happiness index, a culturally sensitive indicator developed by the Korean Psychological Association [19]. Happiness has different meanings according to an individual’s view of life or values, and even the same person has a different point of view of happiness depending on his/her situation. The Korean happiness index is comprised of 3 subdomains: life satisfaction (the cognitive element of happiness, 3 items), positive affectivity (3 items) and negative affectivity (3 items). In this study, all items were rated on a Likert-type scale, ranging from strongly disagree (1) to strongly agree (7). Item scores were summated for each subdomain. The happiness score was calculated by subtracting the negative affectivity score from the combined score of life satisfaction and positive affectivity, which was then transformed into a 100-point scale. Higher scores in life satisfaction, positive affectivity and the overall happiness score indicated a better point of view of happiness, whereas higher scores in negative affectivity indicated an unpleasant emotion. Acceptable reliability and convergent and discriminative validity were demonstrated in a previous study [20]. This instrument was likewise determined to be reliable in our study (Cronbach’s α = 0.75–0.92).

Nursing work environments were assessed using the Korean version of the Practice Environment Scale of the Nursing Work Index [20]. This instrument has been used globally to measure nursing practice environments. It consists of 29 items representing the following 5 subdomains: nurse participation in hospital affairs (9 items), nursing foundation for quality of care (9 items), nurse manager ability, leadership and support of nurses (4 items), staffing and resource adequacy (4 items) and collegial nurse-physician relationships (3 items). Each item has a 4-point Likert scale ranging from 1 (strongly disagree) to 4 (strongly agree). In this study, each subdomain score was calculated using the average score of the items. Higher scores indicated better perceived nursing work environments. Nursing practice environments can be classified as favorable (having 4–5 subdomains with scores higher than 2.5), mixed (having 2–3 subdomains with scores higher than 2.5) or unfavorable (having 0–1 subdomain with scores higher than 2.5) [21]. This scale was demonstrated to have satisfactory construct validity and reliability in a previous study [20]. A reliable internal consistency with Cronbach’s α ranging from 0.67 to 0.83 was found in the present study.

Job crafting was measured using the Korean version of the job crafting questionnaire [22]. This instrument has 3 subdomains with 5 items in each: (1) task crafting, which refers to shaping the nature and scope of the work; (2) cognitive crafting, which involves redefining one’s perception of the job; and (3) relationship crafting, which describes changing the type and nature of interactions with colleagues at work. Item responses range from 1 (strongly disagree) to 5 (strongly agree). Each subdomain was evaluated based on the mean of the item score, with higher scores indicating higher levels of job crafting. The instrument was reported to have concurrent, convergent and construct validity [22] and exhibited good reliability in our study (Cronbach’s α = 0.78–0.85).

Additional participant data collected included demographic characteristics (age, gender, marital status and educational attainment) and job-related variables (years of experience working as a registered nurse, work units, work schedule, overtime, position and monthly income).

### 2.4. Data Analysis

Descriptive analysis involved frequencies and percentages for categorical variables and means and standard deviations for continuous variables. To compare job crafting according to personal and job-related characteristics, analysis of variance and *t*-test were performed. To examine the associations of happiness and nursing work environments with job crafting, we generated multiple linear regression models. The outcome variable, job crafting, was checked for normality using the Shapiro–Wilk test and was found to be normal (*p* > 0.05). The independent variables, happiness and nursing work environments, were treated as continuous variables. The statistical model was adjusted for potential confounding from personal and job-related characteristics with the exception of age (due to its strong correlation with years of registered nurse experience) and gender (few male nurses in the sample). Analyses were conducted using SPSS Statistics for Windows Version 25.0 (IBM Corp., Armonk, NY, USA).

The Institutional Review Board of Seoul National University Bundang Hospital reviewed and approved the study protocol (IRB NO: B-1903-528-301).

## 3. Results

The demographic characteristics of the participants are shown in Table 1. Nurses ranged in age from 23 to 56 years with an average age of 31 years. Almost all of them were female. Approximately two-thirds of the nurses were unmarried and held a bachelor’s degree. The mean years of experience as a registered nurse was 7.5 years with a range of 0.5–25 years. Slightly more than half the participants were working in general wards.

The mean happiness score was 56.8 out of a possible 100 (Table 2). Nurses rated their work environments as mixed (3 subdomains with scores higher than 2.5), with the highest score for nurse manager ability, leadership and support of nurses (2.8) and the lowest score for staffing and resource adequacy (2.0). The mean score of job crafting was 3.6, with cognitive crafting having the lowest score (3.5) and relationship crafting having the highest score (3.7).

Nurses who were satisfied with their lives were significantly more likely to exhibit higher levels of job crafting (B = 0.07, *p* < 0.001) (Table 3). Nursing work environments had no significant association with nurses’ job crafting. In comparison with nurses working in general wards, operating room nurses were significantly less likely to craft their job (B = −0.35, *p* = 0.001).

## 4. Discussion

As job crafters, nurses value the nature of nursing as a profession (i.e., cognitive crafting), develop and use the expertise learned from work experience to improve their practice, embrace new work areas (i.e., task crafting), build intimate relationships with patients and caregivers and interact with colleagues through coaching and mentoring (i.e., relationship crafting) [23]. Our findings indicated that nurses who reported greater satisfaction with their lives were more likely to exhibit proactive attitudes toward crafting their job and roles at work. Life satisfaction is known to create psychological capital, which may facilitate an individual’s ability to think about and interpret their daily lives and events at work in a positive light [24]. Furthermore, those satisfied with their lives could be self-motivated to redesign their jobs [25]. In this case, nurses’ happiness would enhance self-esteem and passion for professional roles and quality job performance [26], which are all associated with job crafting.

Unexpectedly, nursing practice environments had no significant association with nurses’ job crafting when controlling for individual-level happiness in a multivariate statistical model. This suggests that the effect of individual-level happiness on the outcome was stronger than that of organizational level characteristics. Consistent with our findings, job crafting among employees in large Korean corporations has been reported to be associated with individual-level characteristics such as personality, temperaments and autonomy rather than team environmental factors (e.g., team culture and leaders’ leadership style) [27].

In comparison with nurses working in general wards, operating room nurses were less likely to craft their job. Due to the lack of research on job crafting among operating room nurses, the results could not be explained with evidence. Nevertheless, one possible explanation may be differences in job autonomy and decisional authority, which were reported to help workers redesign their job and exercise job crafting in nursing and other professions [28]. In the controlled environments of operating rooms, the scope of duties for nurses is more standardized and formalized; thus, job autonomy is lower. The delivery of regular job-crafting-related education and training programs to operating room nurses could address this issue. Increasing autonomy in a job creates intrinsic motivation due to the perception that one can control one’s job performance [29]. Organizational cultures that establish nurses’ own scope of duties by granting the nurses greater autonomy in independent decision making should be nurtured.

In terms of the job crafting subdomains, our nurses reported the lowest score in cognitive crafting. The cognitive change to solve problems at work can consequently result in a change in the task and relational aspects, which could affect nurses’ organizational commitment and job satisfaction [3]. In order to enhance the value and meaning of nursing and improve task and relationship crafting, it is necessary to offer training programs that can provide directions for job crafting. Moreover, as the behaviors of the job crafting subdomains are interrelated in a complex manner, it is necessary to improve hospital nurses’ job crafting from an integrated perspective of task, cognition and relationship crafting rather than focusing on specific areas alone.

The level of happiness among our study nurses was moderate, which was similar to the findings of a previous study involving Korean nurses [30]; however, it was lower than that observed in a general population of adults [19]. One of the well-known factors that can affect female workers’ happiness is participation in leisure activities, which can relieve job stress and increase both job and life satisfaction [31]. Many organizations now offer leisure support such as reimbursement for cultural activity expenses, travel programs and social clubs. However, hospital nurses may be limited in their leisure activities because of irregular and/or rotating shift schedules and night shifts [32]. Therefore, strategies to increase happiness among nurses should be thoroughly explored. To ensure sufficient leisure time and increase nurses’ happiness, hospital organizations should consider shortening nurses’ working hours through improved staffing, flexible work schedules and reduced overtime.

Our findings should be interpreted with consideration of the study limitations. First, our nurses were recruited from four hospitals through a convenience sampling process; thus, there are concerns over generalizability. Second, our study was cross-sectional and could not demonstrate causal relationships among variables. Lastly, the self-reported data could be vulnerable to denial and social desirability bias.

## 5. Conclusions

Nurses who are job crafters have greater job satisfaction, provide better nursing care and help hospitals achieve organizational goals [4]. Our study demonstrated that happiness at the individual level rather than nursing practice environments at the organizational level was significantly associated with job crafting among nurses. To improve nurses’ happiness and job crafting, organizational supports should be established, such as leisure activity programs and minimal overtime. In addition, organizations should identify the facilitators and barriers of nurses’ job crafting across positions and unit types and provide various opportunities for education and training to promote job crafting. These organizational efforts could contribute to positive work motivation and create an environment where nurses can be happy especially when they have to be at the workplace for an extended period. Future studies should include larger sample sizes from multiple sites so that representative findings can be obtained. In addition, further studies should suggest evidence-based approaches for improving nurses’ happiness and job crafting.

## Figures and Tables

**Table 1:** Demographic characteristics of study participants.

Variable	Categories	*n* (%)
Age, years	Mean (standard deviation)	30.91 (6.08)
	<30	109 (49.5)
	31–39	89 (40.5)
	≥40	22 (10.0)
Gender	Male	12 (5.5)
	Female	208 (94.5)
Marital status	Single	146 (66.4)
	Married	74 (33.6)
Educational (degree) attainment	Associate’s	41 (18.6)
	Bachelor’s	156 (70.9)
	Master’s/Doctoral	23 (10.5)
Years of experience working as a registered nurse	Mean (standard deviation)	7.50 (5.84)
	<3	59 (26.8)
	≥3 and <5	36 (16.4)
	≥5 and <10	58 (26.4)
	≥10	67 (30.5)
Work units	General wards	118 (53.6)
	Intensive care unit, Emergency room	62 (28.2)
	Operating rooms	23 (10.5)
	Outpatients	17 (7.7)
Work schedule	Fixed	40 (18.2)
	Rotating	180 (81.8)
Overtime, hour(s) per day	<1	79 (35.9)
	≥1 and <2	116 (52.7)
	≥2	25 (11.4)
Position	Staff nurse	185 (84.1)
	Charge nurse	35 (15.9)
Monthly income, million won	<3	60 (27.3)
	≥3 and <3.5	108 (49.1)
	≥3.5	52 (23.6)

**Table 2:** Descriptive findings of key variables.

Key Concept	Subdomain	Mean (SD)	Minimum	Maximum
Happiness	Total score	56.80 (15.49)	0	94.35
	Life satisfaction	14.48 (3.11)	3	21
	Positive affectivity	13.43 (3.54)	3	21
	Negative affectivity	12.21 (3.63)	3	21
Nursing work environments	Total score	2.51 (0.38)	1.24	3.79
	Nurse participation in hospital affairs	2.36 (0.47)	1.33	3.78
	Nursing foundation for quality of care	2.76 (0.35)	1.11	3.78
	Nurse manager ability, leadership and support of nurses	2.77 (0.58)	1	4
	Staffing and resource adequacy	1.96 (0.58)	1	3.75
	Collegial nurse–physician Relationships	2.56 (0.60)	1	4
Job crafting	Total score	3.59 (0.52)	2	4.93
	Task crafting	3.58 (0.56)	1.8	5
	Cognitive crafting	3.50 (0.71)	1	5
	Relationship crafting	3.68 (0.60)	1.8	5
SD—standard deviation				

**Table 3:** Associations of happiness and nursing work environments with job crafting among nurses.

Independent Variable	Categories	B	*p*
Happiness	Life satisfaction	0.07	<0.001
	Positive affectivity	−0.02	0.159
	Negative affectivity	−0.01	0.212
Nursing work environments	Nurse participation in hospital affairs	0.13	0.206
	Nursing foundation for quality of care	0.07	0.529
	Nurse manager ability, leadership and support Of nurses	0.02	0.737
	Staffing and resource adequacy	0.03	0.655
	Collegial nurse–physician relationships	0.09	0.108
Age, years	<30	0.21	0.213
	31–39	0.06	0.614
	≥40	reference	
Gender	Male	0.14	0.248
	Female	reference	
Marital status	Single	−0.14	0.081
	Married	reference	
Educational attainment	Associate’s	−0.04	0.692
	Bachelor’s	−0.12	0.259
	Master’s/Doctoral	reference	
Years of experience working as a registered nurse	<3	−0.30	0.055
	≥3 and <5	−0.03	0.842
	≥5 and <10	0.07	0.505
	≥10	reference	
Work units	Intensive care unit, Emergency room	0.08	0.260
	Operating rooms	−0.35	0.001
	Outpatients	0.08	0.587
	General wards	reference	
Work schedule	Fixed	0.10	0.344
	Rotating	reference	
Overtime, hour(s) per day	<1	−0.18	0.090
	≥1 and <2	−0.14	0.115
	≥2	reference	
Position	Staff nurse	0.07	0.440
	Vharge nurse	reference	
Monthly income, million won	<3	0.05	0.661
	≥3 and <3.5	−0.10	0.337
	≥3.5	reference	
